# Reversible Dysphagia Associated With Risperidone Presenting With a Choking Episode

**DOI:** 10.7759/cureus.42491

**Published:** 2023-07-26

**Authors:** Shradha Pandit, Krishna Mahat

**Affiliations:** 1 Internal Medicine, Bandipur Hospital, Bandipur, NPL; 2 Internal Medicine, Ascension St. John Hospital, Detroit, USA

**Keywords:** dysphagia, risperidone, aspiration pneumonia, antipsychotics, tardive dyskinesia, oropharyngeal dysphagia

## Abstract

Dysphagia has been associated with antipsychotic drug use. This case report describes the management of dysphagia in a psychiatric patient who presented to the emergency department from a psychiatric facility after choking on a hot dog. The patient was on risperidone 4 mg, initiated a month prior to treat acute psychosis. Foreign body removal from the distal trachea was performed by bronchoscopy, followed by a swallow evaluation by the speech and swallow team. The patient exhibited severe oropharyngeal dysphagia, leading to aspiration pneumonia and subsequent enteral feeding through a nasojejunal tube. Changes in medication from risperidone to aripiprazole, along with a short course of benztropine and dietary modifications, were implemented, with gradual improvement in swallowing function observed during the hospital stay. The patient's complex medical and psychiatric history contributed to a prolonged hospital stay.

## Introduction

Dysphagia is defined as difficulty swallowing. People can have difficulty swallowing liquid, solid food, or both. The swallowing mechanism is a complex process involving the coordination of muscles in the mouth and throat. Dysphagia can present as choking or the sensation of food getting stuck in the throat or chest. Neuroleptics can cause extrapyramidal side effects, commonly dystonia, parkinsonism, tardive dyskinesia, and akathisia [1]. Clozapine and risperidone cause the fewest extrapyramidal side effects. Acute dystonia presents within the first few days after starting the antipsychotics, while tardive dyskinesia is seen after months [2]. Dysphagia can occur in psychiatric patients, and its management requires a multidisciplinary approach. We present a case of dysphagia in a psychiatric patient who experienced a choking incident and subsequent complications, highlighting the challenges in diagnosis, treatment, and patient disposition. It is found that the prevalence of tardive dyskinesia ranges from 0.4% to 4% in patients taking neuroleptics [3]. The literature review revealed a few similar case reports about risperidone-induced dysphagia [4-5]. One of the case reports had an interesting finding of sudden-onset dysphagia with uvular enlargement after starting on risperidone [6]. This case is unique due to the degree of dysphagia leading to a choking episode that needed urgent emergency intervention. It is important to recognize dysphagia in patients who are taking neuroleptics, as the effects can be reversible [7]. Even though the risk of extrapyramidal side effects is higher with typical antipsychotics, it is still important to be vigilant about possible side effects and assess the risk of events with an eventual fatal outcome, such as laryngeal dystonia with airway obstruction, after starting atypical antipsychotics.

## Case presentation

**Figure 1:**
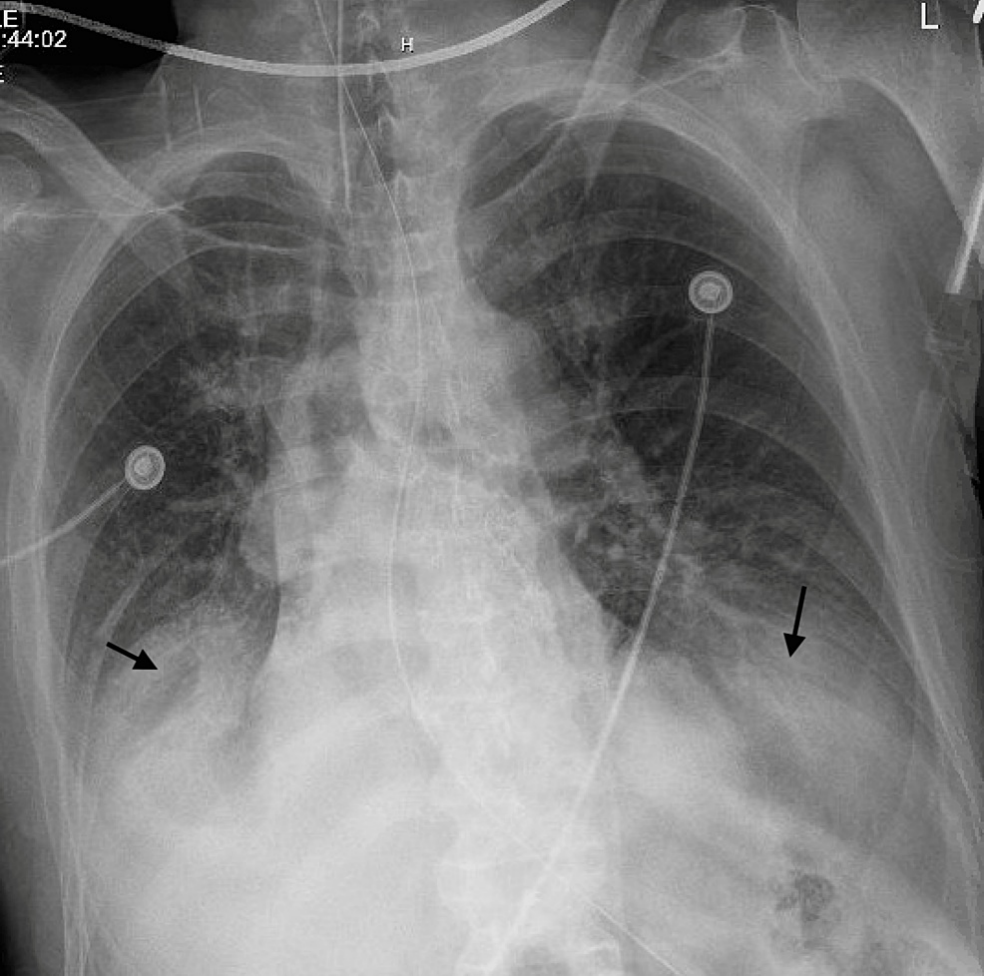
Chest X-ray after endotracheal intubation showing bibasilar atelectasis from airway obstruction.

A 42-year-old male was admitted to the emergency department after choking on a hot dog at a psychiatric facility. The patient was diagnosed with schizophrenia about a month ago and was being treated in an inpatient psychiatric facility. History was limited as no immediate family members were available, and the patient was a poor historian. According to paperwork from the psychiatric facility, the patient was petitioned by some of his family members after he was found urinating in public. He had been receiving risperidone 4 mg tablets for one month after the diagnosis. Besides risperidone, the patient was not on any other medications. The patient did not have any other medical or psychiatric illnesses. There was no history of any other psychiatric medication use. He did not have any issues with swallowing in the past. The patient was in significant respiratory distress upon presentation to the emergency department and later became unresponsive and apneic. The patient was intubated emergently in the emergency department. Bedside airway inspection revealed a piece of hotdog obstructing the distal trachea. The interventional pulmonology and critical care teams successfully removed a piece of sausage, 3.0 cm × 2.5 cm long and wide, respectively, via bronchoscopy in the emergency department. Initial chest X-rays revealed subtle bi-basilar atelectasis (Figure 1).

**Figure 2:**
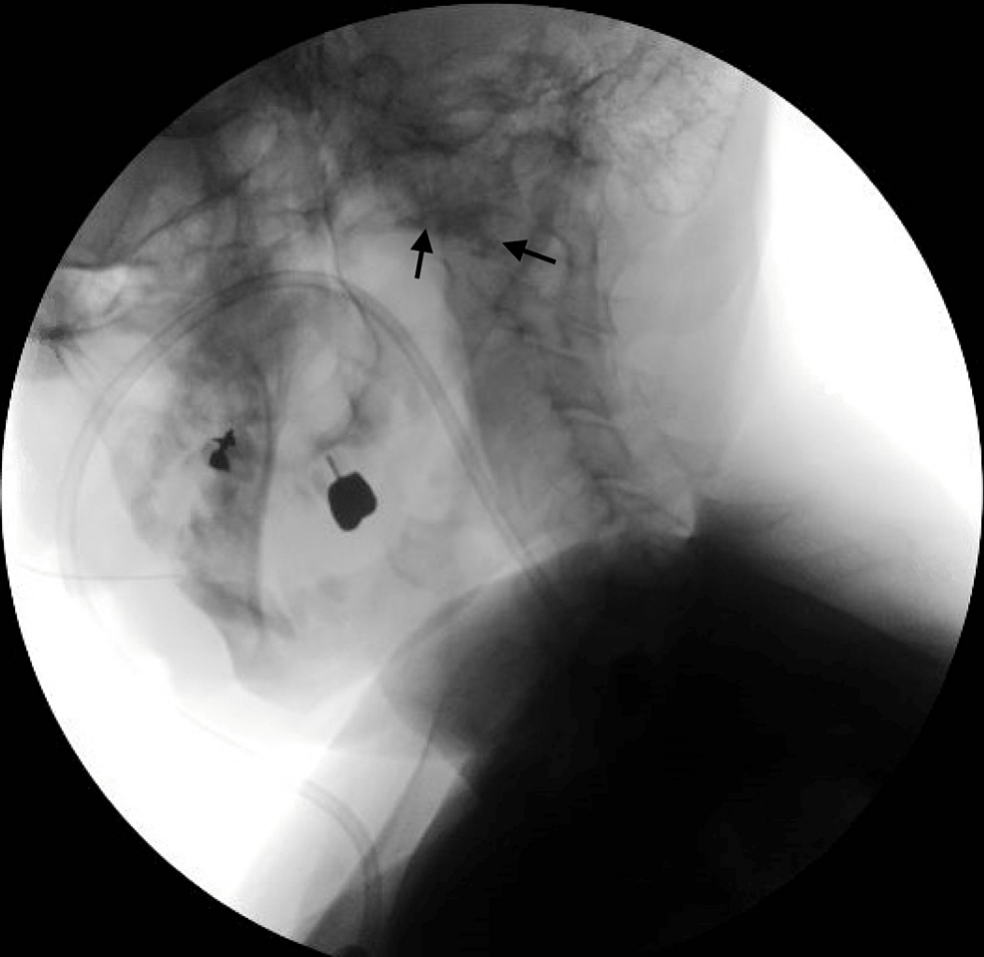
X-ray swallow function with fluoroscopy showing residue present within the valleculae and piriform sinuses.

The patient was extubated successfully soon after the removal of the foreign body. The patient had trouble swallowing intermittently for about two to three weeks. The level of dysphagia according to the adverse drug reaction probability scale was a score of 5 (the drug being the probable cause of the adverse event). The WHO-UMC Causality Category gave probable/likely for risperidone as the cause of dysphagia. Given the significant dysphagia, it was decided to admit the patient for further workup and management of dysphagia. The patient had a very flat face and poor eye contact. No delusions or hallucinations were observed. Examination of the oral cavity did not reveal any uvular or pharyngeal edema. A swallow evaluation by the speech and swallow team was conducted, which showed weak swallowing propulsion with residue in the oropharynx, indicating severe oropharyngeal dysphagia, necessitating the implementation of dysphagia management strategies (Figure 2).

The patient was started on one-to-one feeds with a dysphagia 2 diet and strict aspiration precautions. Unfortunately, the patient developed aspiration pneumonia on day 2, warranting the insertion of a naso-jejunal tube for enteral feeding and a course of antibiotic therapy with ampicillin-sulbactam. Psychiatry was consulted, risperidone was discontinued, and the patient was transitioned to another antipsychotic medication, aripiprazole (2 mg a day). Benztropine was also given for a few days on suspicion of potential extrapyramidal side effects.

On day 10, a repeated speech and swallow evaluation revealed some improvement in swallowing function, leading to the advancement of the diet from naso-jejunal tube feeding to oral feeding with the dysphagia 1 diet. On day 13, further improvement was noted, allowing for the progression of the diet to dysphagia 2.

The patient's poor cognition necessitated continued one-to-one feeding. On day 26, the patients’ diet was advanced to dysphagia 3 with one-to-one feeding with aspiration precautions, including elevation of the head of the bed to 30 degrees (Table 1).

**Table 1:** Levels of national dysphagia diet.

Dysphagia diet level	Description	Examples
Level 1	Pureed (homogenous, very cohesive pudding-like, requiring very little chewing ability)	Pudding, hummus, pureed fruits and vegetables, pureed oatmeal
Level 2	Mechanical altered (cohesive, moist, semisolid foods, requiring some chewing)	Scrambled eggs, mashed potatoes, pancakes, well-cooked vegetables, canned/cooked soft fruits
Level 3	Advanced (soft foods that require more chewing ability)	Bread slices, muffins, moistened cereals, pasta, fish, baked potatoes
Level 4	Regular	No food avoidance or restrictions

The patient's hospital course was complicated by episodes of aspiration pneumonia and COVID pneumonia. He did not require supplemental oxygen or antiviral therapy for the COVID-19 infection. Despite improvements in swallowing function, the patient experienced a prolonged hospital stay due to challenges in finding a suitable group home that could accommodate his complex medical and psychiatric needs. Finally, the patient was discharged to a group home after six weeks.

## Discussion

Dysphagia is not uncommon in psychiatric patients [8]. According to a retrospective study of 31 incidents of dysphagia in 18 psychiatric patients, the most common causes of dysphagia were fast eating and bradykinetic dysphagia [9].

The differential diagnoses we considered in this patient were medication-induced, cerebrovascular accidents, fast eating, and structural abnormalities of the pharynx like Zenker's diverticulum. The type of dysphagia was oropharyngeal, as confirmed by the swallow study. The videofluoroscopy did not reveal any anatomic abnormalities like Zenker's diverticulum. CVA was ruled out due to the absence of other deficits like dysarthria, facial droop, or any weakness of extremities and the absence of other risk factors.

Stewart described a case of risperidone-induced dysphagia in a 76-year-old man with a history of Alzheimer's dementia. He has experienced difficulty swallowing since he started on risperidone. His dysphagia improved once risperidone was discontinued and olanzapine was started [10].

Several studies have reported a significant association between antipsychotic use and dysphagia. According to the systemic review done by Font, the prevalence of patients with swallowing problems taking antipsychotics ranged from 21.9% to 69.5%. In contrast, the prevalence of patients without swallowing problems taking antipsychotics ranged from 5% to 30.5% [11].

The exact mechanisms by which antipsychotics induce dysphagia are not fully understood. However, several potential mechanisms have been proposed. One hypothesis suggests that antipsychotics may lead to decreased salivation, which can affect the swallowing process. Another mechanism could be extrapyramidal side effects causing acute dystonia involving muscles of deglutition. Additionally, antipsychotics may cause sedation and impair the muscles' coordination in swallowing. The anticholinergic effects of antipsychotics can also impair the smooth coordination of the muscles of the pharynx [12]. Another possible mechanism involves the blockade of dopamine receptors, which are present in the basal ganglia and have a role in regulating swallowing movements.

Certain risk factors can predispose individuals to developing dysphagia while on antipsychotic medications. In an observational study done by Anna-Maija et al., the use of antipsychotics was associated with a higher risk of developing pneumonia [13]. Advanced age, underlying neurological conditions, and concomitant use of other medications with anticholinergic properties have been identified as potential risk factors. The clinical presentation of dysphagia induced by antipsychotics can vary, including symptoms such as coughing, choking, regurgitation, and difficulty swallowing solids or liquids.

Dysphagia resulting from antipsychotic use is usually reversible. Discontinuing the culprit medication, lowering the dosage, or changing the antipsychotic drug usually improves dysphagia.

This case highlights the complexities of managing dysphagia in psychiatric patients. Medication adjustments, dietary modifications, and close interdisciplinary collaboration were crucial in addressing the patient's dysphagia. The patient's psychiatric condition, poor cognition, and aspiration risk further complicated the management and prolonged hospitalization.

## Conclusions

Dysphagia in psychiatric patients requires comprehensive evaluation and management. Dysphagia in such a patient population should be evaluated for any potential medication side effects, especially from neuroleptics, which could be life-threatening due to the risk of aspiration. Dysphagia due to antipsychotic use is usually reversible and, in most cases, improves by lowering the dose, discontinuing, and/or changing the antipsychotic medication. Multidisciplinary collaboration, including speech and swallow evaluation, medication adjustments, dietary modifications, and careful monitoring, is essential to optimizing swallowing function and reducing the risk of aspiration.
